# Do home mathematical activities relate to early mathematical skills? A systematic review and meta‐analysis

**DOI:** 10.1111/cdev.14162

**Published:** 2024-10-03

**Authors:** Ella James‐Brabham, Claudia C. von Bastian, Carmel Brough, Emma Blakey

**Affiliations:** ^1^ Centre for Early Mathematics Learning Loughborough University Loughborough UK; ^2^ Department of Psychology University of Sheffield Sheffield UK

## Abstract

Children's foundational mathematical skills are critical for future academic attainment. While home mathematical activities (HMAs) have been proposed to support these skills, the extent to which engaging in them supports mathematical skills remains unclear. This preregistered systematic review and multilevel meta‐analysis identified 351 effect sizes from 72 samples in 20 countries, exploring the relation between frequency of HMAs and mathematical skills in children aged 7 years and under (*M*_age_ = 61 months). A small significant positive relation was found (*r* = .13), moderated by risk of bias, with larger effects associated with a higher risk of bias. Specific ways the field can move forward are discussed to better understand the role of the home mathematical environment in early mathematics.

**Abbreviations**
HMA: home mathematical activity
PRISMA: Preferred Reporting Items for Systematic Reviews and Meta‐Analyses
SES: socioeconomic status

Children's mathematical skills rapidly develop in the preschool years. However, attainment gaps are already visible by age 4 (Blakey et al., 2020; James‐Brabham et al., 2023; Sirin, 2005). To support the development of children's mathematical skills and narrow disparities, identifying factors amenable to intervention early in development is crucial (Every Child a Chance Trust, 2009; Shonkoff et al., 2000). The home environment has emerged as a promising factor, as this is where children spend a large proportion of their time before they start school (Byrnes & Wasik, 2009). The home environment has the potential to provide numerous opportunities for learning (Bronfenbrenner, 1979; Vygotsky, 1978). Its influence may be indirect, for example, through parental scaffolding, learning resources, and parent attitudes to mathematics, or more direct, such as through activities that have mathematical learning embedded, like home mathematical activities (HMAs). Research aiming to understand the role of HMAs in children's mathematical skills is a relatively new field. So far, the strength of the relation between HMAs and mathematical skills varies widely within the literature. These inconsistencies make it difficult to draw conclusions about whether HMAs are a promising way to support children's mathematical development. The current systematic review and multilevel meta‐analysis aimed to synthesize current findings to determine the strength of the relation between HMA frequency and early mathematical skills, and identify factors that influence the strength of this relation.

A wealth of literature supports the broad notion that the home learning environment supports the development of academic skills (Lehrl et al., 2020). However, until recently, relatively less attention has been paid to the role of HMAs in the development of early mathematical skills (Hornburg et al., 2021). Over the past decade, there has been rapid growth in studies exploring the role of the home mathematical environment for mathematical skills. Most studies exploring the home mathematical environment have focused on the *frequency* of parental engagement in HMAs with their children (Daucourt et al., 2021). This is based on the rationale that the more frequently parents engage in HMAs with their children, the more opportunities there will be for children's mathematical skills to be nurtured. Frequency of HMAs is commonly measured by asking parents to retrospectively indicate (e.g., within the past month) how frequently they engaged in a predetermined list of HMAs using a five‐point Likert scale which often ranges from “activities did not occur” to “activity occurred almost daily” (e.g., Cahoon, Gilmore, et al., 2021; DeFlorio & Beliakoff, 2015; Lefevre et al., 2009; Skwarchuk et al., 2014; Sonnenschein et al., 2012). There is wide variation in the frequency of HMAs parents' reports (DeFlorio & Beliakoff, 2015; Lefevre et al., 2009). Thus, if HMAs are important for early mathematical development, they may help explain the substantial variation seen in children's mathematical skills prior to the start of formal education, and may provide a promising target to reduce these disparities.

Several studies have found a relation between the frequency of HMAs and mathematical skills (e.g., Lefevre et al., 2009; Skwarchuk et al., 2014). However, the findings of the literature have been inconsistent, with some studies reporting no significant relation (Cahoon, Gilmore, et al., 2021; James‐Brabham et al., 2023; Missall et al., 2015; Zhou et al., 2006) and others a *negative* relation (e.g., Blevins‐Knabe et al., 2000; Ciping et al., 2015). These inconsistent results between studies make it difficult to draw firm conclusions and are further problematic because studies that do find a positive relation often call on future research to develop interventions on HMAs to support children's mathematical skills (Skwarchuk, 2009). However, this may be premature if the relation is very weak, is negative, or can be explained by other unmeasured variables. Therefore, before any recommendations can be made to caregivers, we need to first determine if there is a relation, and then further explore whether this is a causal relation, and if so, which activities are most impactful through carefully designed randomized control trials.

Inconsistencies in the results in the literature exist not only between studies but also *within* studies. For example, Lefevre et al.'s (2009) seminal study was one of the first key studies to highlight a relation between the frequency of HMAs and mathematical skills. However, mixed findings are reported even within Lefevre et al.'s (2009) seminal study. The study reports eight different relations between HMAs and children's mathematical skills (number skills, number books, games, applications) and mathematical skills (knowledge, fluency). Of these associations, only the correlations between the home mathematical games and the mathematical knowledge and fluency were significantly positive. Markedly, the number books and mathematical fluency were significantly negatively related. The five other correlations reported between HMAs and mathematical skills were not significant. Several other studies report similar internal inconsistencies in results (e.g., Ciping et al., 2015; del Río et al., 2017; Huang et al., 2017; Ramani et al., 2015), making it difficult to draw conclusions about the strength of relation between HMAs and early mathematical skills.

These inconsistencies may be explained by study and sample characteristics. First, it is possible that sample characteristics such as children's age, family socioeconomic status (SES), and geographical location influence the strength of the relation. For example, frequency and types of HMAs change even over the preschool period (DeFlorio & Beliakoff, 2015). It is conceivable that HMAs have a greater influence on mathematical development in early childhood, as this is when children primarily receive mathematical learning input from adults. During this stage, children require guidance and support to build their knowledge and skills, and parents play a key role in scaffolding this learning. In contrast, older children are more capable of directing their own learning and are influenced by a wider range of factors beyond the home environment as they start school and build stronger relationships with peers. Relations between home mathematical learning and children's mathematical skills are larger in families from higher SES backgrounds (Dunst et al., 2017), and the type and frequency of HMAs vary across countries, for example, Canadian parents reported a higher frequency of making or sorting collections and using computer software, whereas Greek parents reported higher frequency of playing board or card games (LeFevre et al., 2010). Cultural beliefs and practices that may vary according to family SES and the country in which they reside in are likely to shape the frequency and type of mathematical activities parents engage in. For example, wide‐ranging early‐year provisions may support parents and equip them with the knowledge to implement home learning activities, compared to parents who have less access to such support (Bertram et al., 2016; Organisation for Economic Co‐operation and Development, 2019).

Second, study characteristics may affect the strength of relations found, such as the study design (cross‐sectional or longitudinal) and measures used. For example, HMAs may lay important foundations for later mathematical skill development by providing building blocks for more advanced mathematical skills. Therefore, longitudinal studies may show stronger effects as they capture growth over time. The huge variation in how we measure HMAs and early mathematical skills may also explain disparities in the findings. To elaborate, some studies differentiate specific HMAs within a questionnaire into formal or direct activities, where teaching mathematics is intentionally targeted (e.g., number cards), and informal or indirect activities, where mathematical learning is not the central focus but may happen incidentally (e.g., board games). Other studies make no distinction between types of activities within the questionnaire; thus, it is likely the way activities are conceptualized and analyzed contribute to differences in results. In reference to mathematical measures, there is substantial variety in the type of measures studies use. While composite measures tap into a range of skills, recently, Gilmore (2023) and Devlin et al. (2022) have highlighted the importance of considering mathematical skills separately that contribute to overall mathematical achievement, as they are likely to have distinct influences. Within the HMA literature, studies have used composite or standardized measures of mathematical achievement to capture multiple skills within a single outcome score, and other studies use specific measures of individual mathematical skills (e.g., rote counting, cardinality, numerical magnitude, operations, spatial skills, and nonsymbolic measures). It is possible that not all HMAs have equal influence on all mathematical skills, and that specific HMAs may relate more strongly to specific mathematical outcomes, for example, we may not expect HMA frequency relating to number to influence spatial skills (Andrews et al., 2021; Gilmore, 2023).

Third, studies may vary in their risk of bias, which could cause inconsistencies in the field. Studies that have used less valid and reliable measures or underpowered samples are more likely to show unreliable or nonreplicable effects (Higgins et al., 2023). At present, we do not know how prevalent risk of bias is in the field.

Understanding the role of HMAs for early mathematical development is important because it offers a possible target for intervention if there is a genuine relation. Here, perhaps lessons can be drawn from research on children's literacy. Large effect sizes have been observed between frequency of home literacy activities and literacy skills (Dong et al., 2020), and these home practices have been shown to be malleable to intervention (Sénéchal & Young, 2008). Therefore, it may be possible to also intervene and increase the quantity and quality of HMAs using similar approaches. Home learning activities could be targeted in two ways: first, by equipping parents with the understanding that HMAs are beneficial, the knowledge of the types of mathematical activities to engage in, and how to engage in them, and second, by giving parents the resources to engage in home activities, for example, books (National Literacy Trust, 2018). Within literacy interventions, both approaches together have been found to be effective (de Bondt et al., 2020; Save the Children, 2016). However, before interventions are developed for HMAs, it is crucial that we establish whether there is an overall relation between HMAs and mathematical skills.

## Prior reviews

A handful of previous reviews synthesized the home mathematical environment literature. Two of these reviews aimed to *qualitatively* synthesize the literature on frequency of HMAs and mathematical skills. Bennett (2017) and Mutaf‐Yıldız et al. (2020) narrative reviews highlighted significant inconsistencies in the literature and suggested a range of factors, including children's age and type of measures used, that may explain inconsistencies in the field. Only a few quantitative systematic reviews and meta‐analyses are available, and all investigated the home mathematical environment more broadly rather than focusing on HMA frequency specifically. Dunst et al.'s (2017) meta‐analysis examined the relation between family numeracy learning experiences and mathematical skills in 13 samples of children between 3 and 7 years of age and reported an overall effect size of 0.46. However, this meta‐analysis did not include key studies such as Lefevre et al.'s (2009) seminal study but, given its broader scope, it did include studies which had literacy questions within the activity questionnaire. A more recent meta‐analysis across 65 studies by Daucourt et al. (2021) found a small positive relation between the home mathematical environment and mathematical skills (*r* = .13). This review conceptualized the home mathematical environment not only as frequency of activities but also as number talk and parent attitudes and beliefs about mathematics. This all‐encompassing approach enabled an understanding of how the home mathematical environment more broadly relates to children's mathematical skills and the factors that moderate this broad relation. However, while this review did explore the strength of relation between HMA frequency and mathematical skills, it was not able to look at the factors that specifically moderate the relation between frequency of HMA's and mathematical skills.

## The present study

This preregistered systematic review and multilevel meta‐analysis aimed to fill three gaps in the literature. First, as yet, it is unclear whether HMA frequency is positively related to mathematical skills and whether there are factors that moderate this relation. Second, given the recent rapid increase in studies exploring HMA frequency over the last few years, 19 new eligible studies have been published since Daucourt et al.'s (2021) review, where the literature search was conducted in 2018. Third, past reviews have not systematically and quantitatively assessed risks of bias. The aim of the current meta‐analysis is to provide a quantitative synthesis of existing research which has explored the relation between frequency of HMAs and early mathematical skills in young children, addressing three research questions: (1) Is there a relation between frequency of HMAs and mathematical skills? (2) How strong is this relation across studies? (3) Can the heterogeneity be attributed to the following moderators: (i) age of children, (ii) SES, (iii) study design (concurrent vs. longitudinal), (iv) type of mathematical skills measure, (v) type of HMA measure (exploratory analysis), (vi) the country of data collection, (vii) publication bias, and (viii) risk of bias? While we did not have specific hypotheses, as the aim was to resolve discrepancies in the field, our choice of moderators was guided by our predictions that these may significantly explain the variation in findings.

## METHOD

The current meta‐analysis was conducted following PRISMA (Preferred Reporting Items for Systematic Reviews and Meta‐Analyses) guidelines. The meta‐analysis was preregistered (https://osf.io/4ubw9/?view_only=251b253fcecd4b4e8a64da62832ebe6b). Any deviations from the preregistration are described below and explained why this was necessary.

### Study selection, data extraction, and coding

#### Literature search

The initial literature search was conducted in June 2019 using three databases: Scopus, Web of Science, and PsycINFO (prior to the Daucourt et al., 2021 review). The search was updated using the same databases in April 2021 as many more papers had been published, and we wanted to ensure that the review was comprehensive and included the new literature. In addition, ProQuest was searched in May 2021 to include student theses and dissertations. The following search terms were used:

(“Home learning environment*” OR “Home numeracy environment*” OR “Home math* environment*” OR “Home learning” OR “Home math* learning” OR “Home numeracy learning” OR “Home learning activit*” OR “Home numeracy activit*” OR “Home math* activit*” OR “Home experience*” OR “Home math* experience*” OR “Home numeracy experience*”)

**AND** (“Math*” OR “Numeracy*” OR “Arithmetic*” OR “Number*”)

**AND** (“Child*” OR “Toddler” OR “Infan*” OR “Pre‐school” OR “Pre school” OR “Preschool*” OR “Kindergarten” OR “Pre‐kindergarten” OR “Pre kindergarten”)

The database search results were exported to EndNote (The EndNote Team, 2013). Reference lists of the final sample of articles were searched to identify relevant articles which had not been located via the database searches, resulting in the inclusion of one further study. In May 2021, we sought out any unpublished data by contacting authors in the field, as well as emailing relevant mailing lists (cogdevsoc, dev‐europe, and mathlink). This resulted in a total of 12 unpublished samples being including in the final meta‐analysis.

### Inclusion criteria

All studies included in the meta‐analysis were required to meet the following criteria:

- Children with a mean age of up to and including 7 years.
- Frequency of HMAs measured using a multi‐item (i.e., more than one item) questionnaire to measure frequency of HMAs.
- A direct measure of child mathematical skills included in the study (i.e., not parent or teacher rated).
- Children must not have known developmental disorders or special educational needs.
- Manuscript must be written in English.
- Study must look at the relation between frequency of HMAs and mathematical skills.

Note that the final inclusion criteria implemented differed slightly from the preregistered inclusion criteria in that the preregistered criteria stated that only published studies would be included. However, to limit publication bias, we decided it was important to expand our inclusion criteria to PhD theses and gray literature.

### Study selection

**FIGURE 1:**
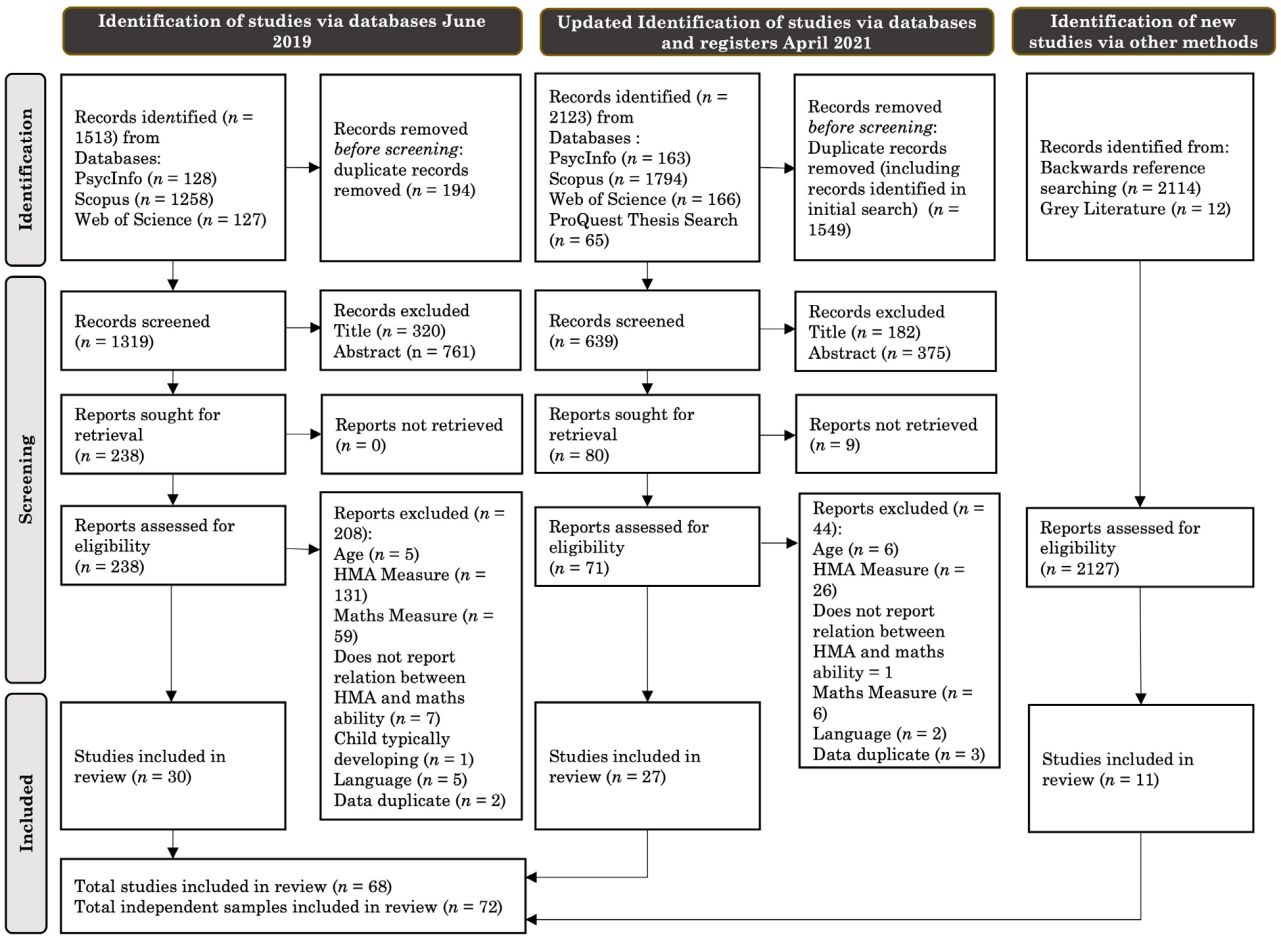
PRISMA diagram detailing the records identified, screened, and included in the meta‐analysis based on the search strategy. HMA, home mathematical activity.

A flow chart depicting the search process and exclusion of studies is displayed in Figure 1. Duplicates were excluded in EndNote based on Bramer et al.'s (2016) procedure. Three trained individuals screened all nonduplicate studies against the inclusion criteria with the lead author checking the screening to ensure accuracy. First, article titles were screened. During title screening, studies that were not within the topic area were excluded. Second, abstracts were screened. During abstract screening, studies which clearly did not meet the inclusion criteria were excluded. The initial search resulted in 238 papers being retrieved for full‐text screening, while the updated search resulted in an additional 71 papers being retrieved for full‐text screening. During full‐text screening, articles were reviewed against the full inclusion criteria. Only articles which met the full inclusion criteria were included in the final meta‐analytic sample. A total of 72 independent samples met the inclusion criteria and constituted the final meta‐analytic sample. To avoid biases from including a single data set multiple times (Borenstein et al., 2009), we included only the published record where both unpublished and published studies were available. If a study included multiple independent samples, these were included and treated as separate studies (Borenstein et al., 2009).

### Data extraction

The following information was extracted from all studies which met the inclusion criteria: (a) bibliographic information (e.g., author, title, year, publication status), (b) study design (i.e., concurrent or longitudinal), (c) sample descriptors (e.g., age, SES information, country of data collection), (d) predictor details (i.e., type of HMA questions used), (e) outcome details (i.e., type of mathematical skills measure), (f) outcome data (i.e., sample size, zero‐order correlation coefficient), and (g) information to assess risk of bias (i.e., instrument reliability, addressing missing data, task drop out, instrument quality, floor or ceiling effects).

If a study met the inclusion criteria but did not report zero‐order correlations between frequency of HMAs and mathematical skills, the corresponding author was contacted via email to procure the data. If authors did not respond to the initial request, authors were sent a follow‐up request 2 weeks later. If authors did not respond to the second request, the study was removed from the meta‐analysis. This resulted in the removal of eight studies.

### Second coding

Data extraction was shared between the lead author, the third author, and a trained research assistant. Eighty‐three percent of independent samples were double coded by the lead author and the third author across the variables: country of data collection (97% agreement), study design (100% agreement), type of mathematical skill measure (92% agreement), and type of HMAs measure (93% agreement). Disagreements were resolved through discussion between the first and third authors, where uncertainty still existed, the last author made the final decision.

### Moderators

Table 1 provides a complete list of moderator variables examined. We developed a more detailed categorization for mathematical skill measure than initially preregistered. This was in response to exploring the type of measures used in the studies in our sample, as well as the theoretical literature (Devlin et al., 2022; Gilmore, 2023). While we did not specify that we would look at SES in the preregistration, our interests developed such that we wanted to examine this. However, while we then intended to test SES as a moderator, inadequate reporting of SES information in most studies meant that SES could not be coded or could not be meaningfully compared, particularly across countries. Inadequate reporting included not reporting any SES information (e.g., Huang et al., 2017) or arbitrarily splitting SES into “high” and “low” categories based on the SES distribution in the study sample rather than based on population SES (e.g., DeFlorio & Beliakoff, 2015; Lefevre et al., 2009).

**TABLE 1:** Moderator variable description.

Variable	Description	Coding
Age of children	This details children's age in months	Continuous measure
Country of data collection	This details the country in which data were collected	
Study design	Details whether the study collected outcome data at a single time point or multiple time points	Concurrent Longitudinal
Type of mathematical skills measure	This details the type of mathematical skills outcome measure taken	Composite Rote counting Number knowledge (e.g., cardinality, mapping, transcoding) Magnitude estimation (e.g., number to position, number comparison) Operations (e.g., addition, subtraction) Spatial skills^a^ (e.g., spatial transformation) Nonsymbolic skills (e.g., dot comparison)
Type of home mathematical activity (HMA) measure	This details the type of HMA measure used in the study	1 = overall 2 = formal (direct) 3 = informal (indirect) 4 = other^b^
Publication status	Details whether the study was located in journal or from another location (e.g., thesis/dissertation/unpublished data)	Published Not published

^a^ Not explored as a moderator as too few studies reported using this measure.

^b^ Other refers to activities that were categorized into categories other than “formal” and “informal,” for example, spatial activities.

When looking to explore country of data collection as a moderator, many countries did not have more than 10 samples (Fu et al., 2010; Higgins et al., 2023). Therefore, to explore this question, we grouped countries into their geographical continental region. These were North America, Europe, Central and South America, and Asia. Table 2 provides details of country grouping. For countries which had more than 10 effect sizes, we explored the strength of relation and whether this significantly moderated the relation (this is reported in Supporting Information S3). We did not preregister these geographical groupings.

**TABLE 2:** Geographical breakdown of country of data collection detailing number of independent samples (*k*) and number of effect sizes (*n*).

Geographical region grouping	Country of data collection	*k*	*n*
North America	The United States	25	84
	Canada	6	22
Europe	Belgium	4	20
	Finland	1	4
	Germany	5	14
	Greece	2	8
	Italy	1	1
	Lithuania	1	6
	The Netherlands	2	2
	Spain	1	6
	The United Kingdom	8	46
Central and South America	Chile	2	22
	Mexico	2	4
Asia	China	3	35
	Hong Kong	2	24
	Philippines	2	12
	Vietnam	1	8
Uncategorized	Australia	2	5
	Russia	1	6
	Turkey	1	1
	Turkey and Canada	1	5

*Note*: Uncategorized countries are countries that did not fit into a geographical region as they were either (i) a mixed sample across countries or (ii) not enough studies or effect sizes to analyze it.

For the type of mathematical skills measure, only three samples reported spatial skills, and thus, we refrained from exploring spatial skills as a moderator.

Following recommendations from reviewers, we also included type of HMA measure as a moderator as an exploratory analysis. We categorized HMA scales as “overall,” “formal,” and “informal” based on the study authors' labeling of activities rather than our own definitions for two reasons: first, not all studies published the questionnaire questions, and second, there is no clear and consistent definition in the literature regarding which activities are considered formal and which are informal (see Andrews et al., 2021).

### Analyses

#### Effect size calculation

Data were analyzed with R (version 4.1.2). Most studies included used correlational designs that report Pearson correlations, and therefore, zero‐order correlation coefficients were used to calculate effect sizes between frequency of HMAs and mathematical skills. A small number of studies used experimental designs (e.g., Niklas, Cohrssen, et al., 2016; Niklas, Nguyen, et al., 2016). In these cases, only baseline data were included. Fisher's *Z*‐transformed correlations and variance of each effect size were calculated using the correlation coefficients and sample size with the function “escalc()” in the package metafor (Viechtbauer, 2010). All analyses were performed with the Fisher's *Z*‐transformed effect size and were then converted back to *r* for reporting.

#### Variability in effect sizes across studies

The average weighted correlation between frequency of HMAs and mathematical skills was estimated using a random‐effects model. A random‐effects model was chosen as the true effect size was expected to vary across studies, whereas a fixed‐effects model would assume that all studies shared a true effect size (Borenstein et al., 2009). We expected variation in effect size to vary across studies because the studies were conducted in different countries, with children of different ages, using different HMA scales, and different measures of mathematical skills.

#### Heterogeneity of effect sizes

Heterogeneity of effect sizes was tested using a *Q* test. *I* ^2^ was then calculated to quantify the proportion of variance in effect size due to heterogeneity (Higgins & Thompson, 2002). Heterogeneity supports the use of a random‐effects model, whereas homogeneity would support the use of a fixed‐effects model.

#### Accounting for dependent effect sizes

Most studies (82%) included in the meta‐analysis reported more than one effect size. Therefore, to allow for estimating unbiased standard errors, a three‐level meta‐analysis clustering effect sizes at both study‐level and observation‐level was run. Study‐level and observation‐level estimates enable the identification of how much variance is a result of between‐study differences (i.e., how the relations differ across studies), compared to within‐study differences (i.e., how the relations differ within a single study).

#### Analyzing variability in effect sizes

To establish whether the moderators explained variance in effect sizes, multiple models controlling for between‐ and within‐study variance were tested. Omnibus tests based on the *F*‐distribution were conducted for each moderator variable. This indicates whether the subgroup effect sizes significantly differ from one another, thus establishing a moderator. Random‐effects multilevel models for each moderator subgroup were conducted to determine the overall effect sizes for each subgroup within a moderator.

#### Evaluation of risk of bias

Risk of bias refers to the possibility that the results of a research study may be distorted due to design and methodological flaws, conduct, or analysis, with high risk of bias potentially affecting the validity and reliability of findings (Hoffmann et al., 2017). Conducting a risk of bias analysis for studies within the review facilitates the critical evaluation of the quality of research included in the review. The current review adapted two risks of bias assessments developed by Hoffmann et al. (2017) and Hjetland et al. (2017) considering key issues related to potential bias in the measures, design, and sample. The quality of these studies was rated across seven dimensions (see Table 3). The lowest possible risk of bias score for a study was 0, and the highest possible risk of bias score was 11.

**TABLE 3:** The risk of bias dimensions and scoring.

Risk of bias dimension	Risk of bias scoring (0, 1, or 2)
Sample size	Above 150 (0)
	70–150 (1)
	Below 70 (2)
Instrument quality: mathematics assessment	Only standardized (0)
	Combination of standardized and unstandardized (1)
	Researcher made (2)
	Not clear (2)
Test reliability: mathematics assessment	Reports on all measures and all reliable: *α* > .6 (0)
	Reports for some measures and all reliable (1)
	Measures not reliable (2)
	Reports from test manual (2)
	Does not report (2)
Test reliability: home mathematical activity scale	Reports on all dimensions and all reliable: *α* > .6 (0)
	Reports on some dimensions and all reliable (1)
	Scale not reliable (2)
	Reports from prior research (2)
	Does not report (2)
Floor/ceiling effects on mathematics assessment	No floor/ceiling effects^a^ (0)
	Floor/ceiling effects (1)
	Does not report (1)
Task drop out on mathematics assessment	Reports drop out: less than 20% (0)
	Reports drop out: more than 20% (1)
	Does not report drop out (1)
Missing data	Better than listwise deletion (i.e., does not remove data cases where one data point is missing) (0)
	Listwise deletion (1)

^a^ The mean value plus/minus the standard deviation did not result in a value below the minimum possible score or above the maximum possible score.

#### Evaluation of publication bias

Publication bias refers to the phenomenon that studies which report significant findings (*p* < .05) are more likely to be published than those without significant findings (Rosenthal, 1979). Publication bias can affect the validity of a meta‐analysis, resulting in an overestimation of the true population effect size and an underestimation in heterogeneity (Lin & Chu, 2018). Publication bias was assessed using visual and statistical techniques.

In addition, a *p*‐curve analysis was conducted to indicate whether there was evidence of *p*‐hacking. *p*‐hacking refers to the phenomenon where researchers engage in practices which make their results more likely to be statistically significant. This can include the way data are collected, analyzed, and selected for reporting (Head et al., 2015). *p*‐curve analysis calculates *pp*‐values, which are the probability of obtaining each *p*‐value if there was no significant effect. To test whether a *p*‐curve is significantly skewed, *pp*‐values were added together to create a *χ* ^2^ value to test the significance of the *p*‐curve skew. A flat *p*‐curve signifies equal probability of observing *p*‐values; a right‐skewed *p*‐curve indicates a true effect and thus a low chance of publication bias. A *p*‐curve with left skew shows that the probability of high *p*‐values is greater than the probability of low *p*‐values and thus demonstrates that there is evidence of *p*‐hacking. *p*‐curve analysis was conducted using the *p*‐curve application available at http://www.p‐curve.com/app4/.

Funnel plots were used to visually assess whether there is publication bias (Light & Pillemer, 1984). Funnel plots show the relation between effect size and standard errors. Effect sizes which are symmetrically distributed around the vertical line indicate no publication bias, whereas effect sizes which are not symmetrically distributed around the vertical line would indicate potential publication bias.

Finally, fail‐safe N was calculated using the Rosenthal method (Rosenthal, 1979). This calculated how many non‐23 significant effect sizes would be required to reduce the combined significance level. Specifically, alphas of *p* < .05 and *p* < .01 were tested.

## RESULTS

### Final sample

The final article sample for the meta‐analysis consisted of 334 effect sizes from 72 independent samples, reported in 68 articles. Each study contributed between 1 and 24 effect sizes (median = 3), with a mean of 288 participants per effect size (SD = 566). The mean age of children across effect sizes was 61 months (SD = 13 months). A summary of study data included can be found in the Supporting Information (S1).

### Overall average weighted correlation between HMAs and mathematical skills

**FIGURE 2:**
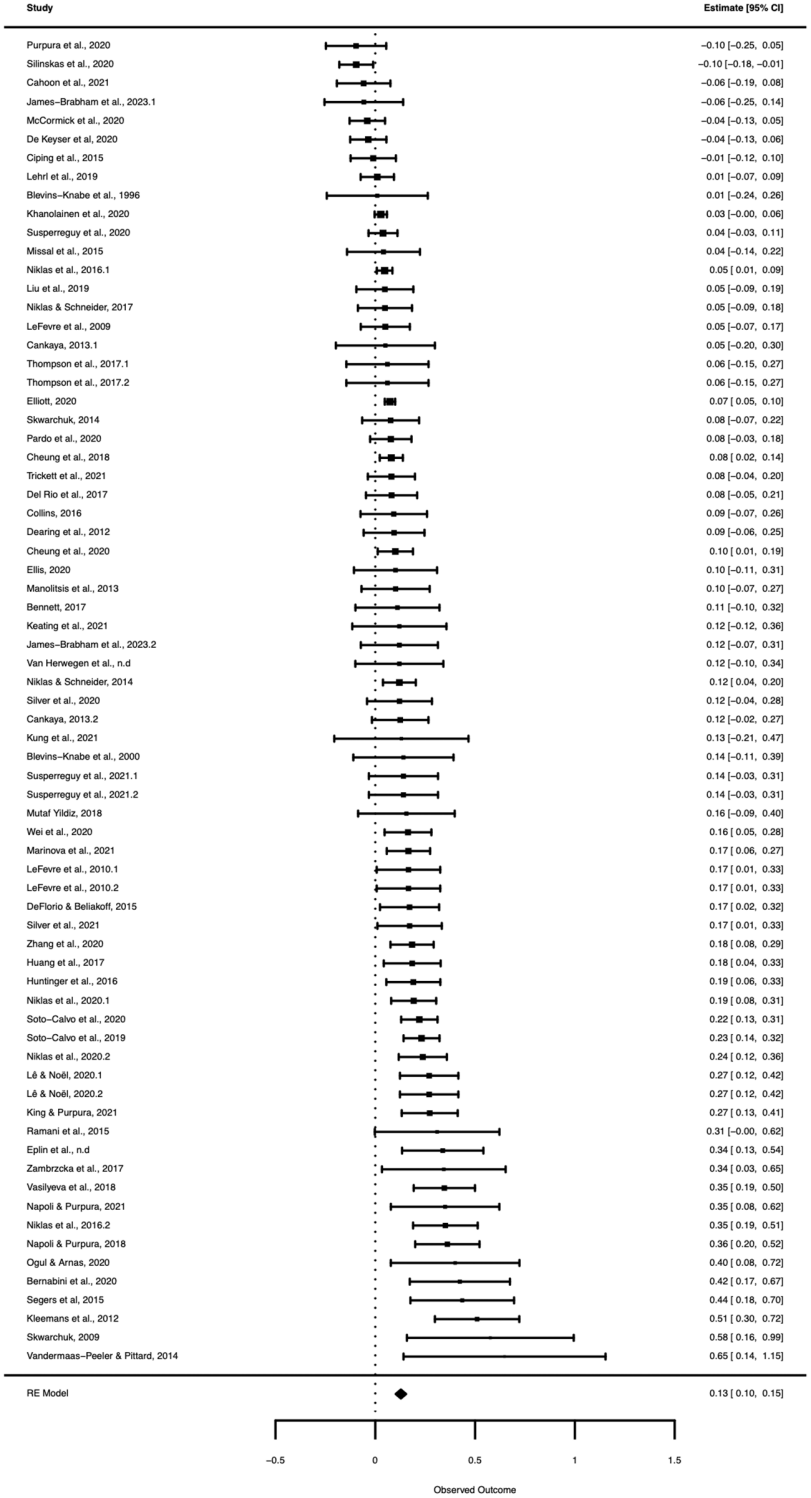
Forest plot depicting average effect size and 95% confidence intervals for each independent sample included in the meta‐analysis. Note that this shows average effect size, where if a study contained more than one effect size, a composite variable with the average effect size was created. For the multilevel meta‐analysis reported, individual effect sizes and not composite effect sizes are used. RE, random‐effect.

The three‐level meta‐analytic model revealed a small significant positive correlation between HMAs and mathematical skills of *r* = .13 (95% CI: [.10, .16], *p* < .001). There was significant variance in the overall average effect sizes, *Q*[333] = 1210.41, *p* < .001, $I_{\text{Total}}^{2}$ = 81.15%, thus supporting the decision to use a random‐effects model that assumes between‐sample variance. Approximately 80% of variance between frequency of HMAs and mathematical skills was not due to sampling error. The estimated variance components were $\tau_{\text{Level}\;3}^{2}$ = .009 and $\tau_{\text{Level}\;2}^{2}$ = .006, with 49% of total variation being attributed to between‐study variation ($I_{\text{Level}\;3}^{2}$) and 33% of total variation was attributed to within‐study variation ($I_{\text{Level}\;2}^{2}$). A three‐level model was compared to a two‐level model where level 3 heterogeneity was constrained to zero. A three‐level model provided a significantly better fit compared to a two‐level model ($\chi_{\left(1\right)}^{2}$ = 77.74; *p* < .001). This supports a three‐level meta‐analysis. Figure 2 shows a forest plot with the composite effect.

### Moderators of the effect

Multiple moderator analyses were conducted to determine whether study design (i.e., cross‐sectional vs. longitudinal), geographical area of data collection, child age, type of mathematical skills measure used, type of HMA measure used, and risk of bias significantly contributed to effect size heterogeneity. Each potential moderator was assessed individually. All moderators were entered as categorical variables, except for age which was entered as a continuous variable. The overall effect size for each subgroup is reported in Table 4. Data were not available for every independent sample across all moderators; therefore, we detail the number of independent samples and effect sizes included in each moderator analysis. Further details can be found in Supporting Information S1 and S2. Country‐specific moderator analysis for countries with more than 10 effect sizes is reported in Supporting Information S3.

**TABLE 4:** Summary of effect sizes for subgroup analyses.

Moderator	Subgroup	*r*	95% CI	*n*	*k*
Geographical location	North America	.14	.09, .19	105	30
	Europe	.12	.07, .16	107	25
	Central and South America	.09	−.03, .21	26	4
	Asia	.13	.06, .20	79	8
Study design	Concurrent	.13	.10, .16	226	65
	Longitudinal	.13	.09, .17	107	24
Type of mathematical skills measure	Composite	.14	.11, .18	164	53
	Rote counting	.09	.03, .16	17	13
	Basic numerical knowledge	.16	.11, .20	57	17
	Numerical magnitude	.09	.03, .15	23	11
	Operations	.12	.07, .18	51	16
	Nonsymbolic	.11	.04, .17	18	11
Type of home mathematical activity measure	Overall	.13	.09, .17	147	39
	Formal	.13	.08, .17	111	31
	Informal	.15	.11, .21	63	20

Abbreviations: CI, confidence interval; *n*, number of effect sizes; *k*, number of independent samples; *r*, Pearson's *r*.

### Moderation effects of age (*k* = 71)

The omnibus test with age was not significant, *F*(1, 331) = 0.18, *p* = .675, $\tau_{\text{Level}\;2}^{2}$ = .01, $\tau_{\text{Level}\;3}^{2}$ = .01, *n* = 332, *I* ^2^ = 81.20%, indicating that the overall relation between frequency of HMAs and mathematical skills was not significantly moderated by age.

### Moderation effects of geographical location of data collection (*k* = 67)

The omnibus test with geographical location of data collection was not significant, *F*(3, 331) = 0.28, *p* = .840, $\tau_{\text{Level}\;2}^{2}$ = .01, $\tau_{\text{Level}\;3}^{2}$ = .01, *n* = 317, *I* ^2^ = 77.72%, indicating that the overall relation between frequency of HMAs and mathematical skills was not significantly moderated by geographical location.

### Moderation effects of study design (*k* = 73)

The omnibus test with study design was not significant, *F*(1, 332) = 0.06, *p* = .800, $\tau_{\text{Level}\;2}^{2}$ = .01, $\tau_{\text{Level}\;3}^{2}$ = .01, *n* = 334, *I* ^2^ = 81.16%, indicating that the overall relation between frequency of HMAs and mathematical skills was not significantly moderated by study design (i.e., whether the effect size was longitudinal or cross‐sectional).

### Moderation effects of mathematical skills measure (*k* = 71)

The omnibus test with mathematical skills measure was not significant, *F*(5, 325) = 1.64, *p* = .150, $\tau_{\text{Level}\;2}^{2}$ = .01, $\tau_{\text{Level}\;3}^{2}$ = .01, *n* = 331, *I* ^2^ = 81.01%, indicating that the overall relation between frequency of HMAs and mathematical skills was not significantly moderated by the mathematical skills measure used.

### Moderation effects of HMA type (*k* = 69)

The omnibus test with HMA type was not significant, *F*(2, 319) = 0.98, *p* = .375, $\tau_{\text{Level}\;2}^{2}$ = .01, $\tau_{\text{Level}\;3}^{2}$ = .01, *n* = 322, *I* ^2^ = 81.42%, indicating that the overall relation between frequency of HMAs and mathematical skills was not significantly moderated by the type of HMA measure used.

### Publication bias

The omnibus test with publication type was not significant, *F*(1, 332) = 0.07, *p* = .797, $\tau_{\text{Level}\;2}^{2}$ = .01, $\tau_{\text{Level}\;3}^{2}$ = .01, *n* = 333, *k* = 71, *I* ^2^ = 81.28%, indicating that the overall relation between frequency of HMAs and mathematical skills was not significantly moderated by publication type. This suggests that published studies are not more likely to report a significant relation between frequency of HMAs and mathematical skills compared to unpublished studies.

**FIGURE 3:**
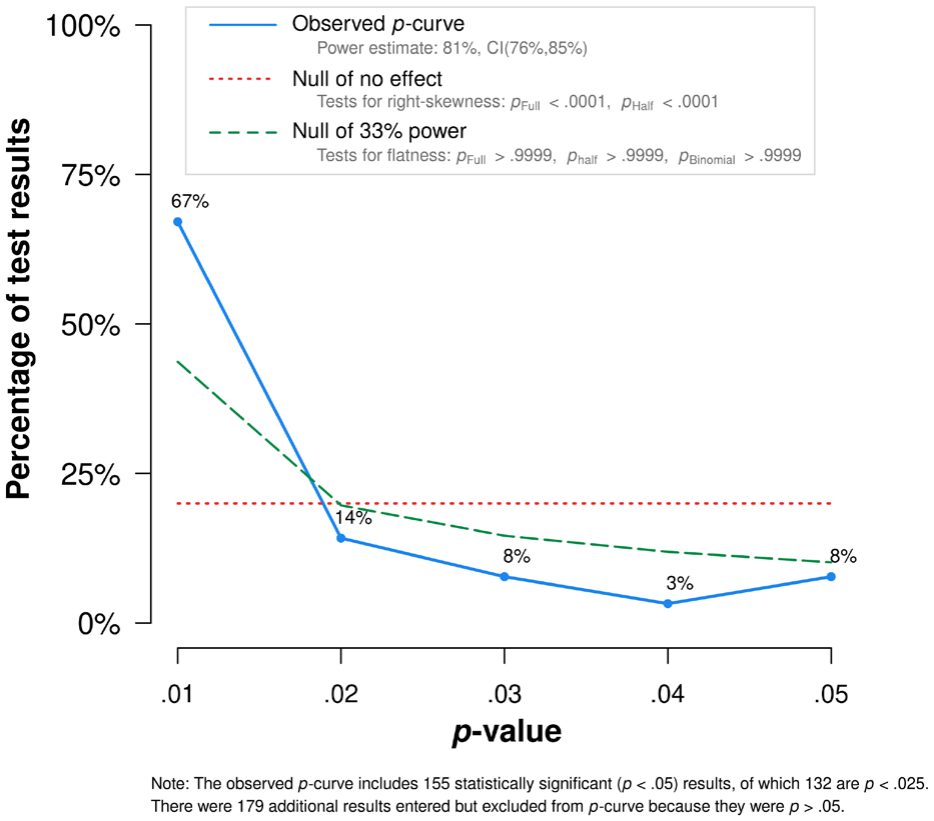
*p*‐curve analysis results which indicate significant right skew, with nonsignificant result for the full‐ and half‐*p*‐curve tests. This indicates no substantial evidence of *p*‐hacking in the meta‐analytic sample.

The *p*‐curve analysis plot is displayed in Figure 3. Both the full, *Z* = −20.00, *p* < .001, and half, *Z* = −17.90, *p* < .001, *p*‐curve test indicated significant right skew. This suggests that it is not likely that the results are caused by publication bias or *p*‐hacking. Furthermore, the full *p*‐curve, half *p*‐curve, and binomial 33% power test were nonsignificant (full: *Z* = 10.56, *p* > .999; half: *Z* = 19.28, *p* > .999; binomial: *p* > .999). Together, these results suggest that there is no evidence of *p*‐hacking in the current meta‐analytic sample.

**FIGURE 4:**
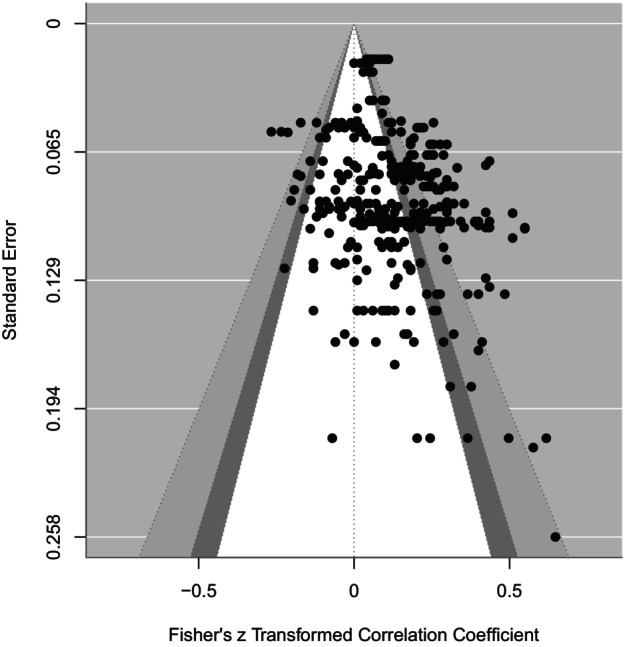
Funnel plot of the multilevel correlated effects meta‐analysis. Confidence intervals on the 90th (white), 95th (dark gray), and 99th (light gray) percentiles.

The funnel plot, displayed in Figure 4, depicts the effect sizes for the meta‐analytic sample relative to their standard errors (Sterne & Egger, 2001). There is some asymmetry in the plot, with a concentration of studies on the right‐hand side. This indicates that publication bias may be present. However, study quality and true effect size can influence the funnel plot.

### Sensitivity analysis

The fail‐safe *N* test using the Rosenthal approach revealed an additional 530,363 effect sizes with a relation of zero would be needed to increase the *p* value to be greater than .01. An additional 101,086 effect sizes with a relation of zero would be needed to increase the *p* value to be nonsignificant (*p* > .05). The large fail‐safe *N* suggests that it is unlikely the results were susceptible to publication bias.

### Risk of bias

We had insufficient information to calculate the risk of bias for five independent samples due to the nature of the gray literature acquired (i.e., results and not methodology were shared with us when acquiring gray literature). Therefore, the risk of bias was calculated for 67 independent samples across 63 studies.

The mean total risk of bias score was 5.64 (SD = 2.04, range 2–9). A summary of the number of studies falling into each bias category across the risk of bias dimensions is shown in Table 5. The full risk of bias coding is displayed within the Supporting Information (S2).

**TABLE 5:** The number of independent samples that fell into each bias category across the risk of bias dimensions.

	Sample size	Instrument quality: Mathematics assessment	Test reliability: Mathematics assessment	Test reliability: HMA scale	Floor/ceiling effects	Task drop out	Missing data
Low risk (0)	23	28	15	46	27	28	26
Higher risk (1)	30	18	7		40	39	41
High risk (2)	14	21	45	21			

*Note*: Sample size, instrument quality: mathematics assessment, test reliability: mathematics assessment, and test reliability home mathematical activity (HMA) scale were coded on a three‐point scale: (0) low risk, (1) higher risk, and (2) high risk. Floor/ceiling effects, task drop out, and missing data were rated on a two‐point scale: (0) low risk and (1) higher risk. Supporting Information S2 contains further details of the coding.

The total risk of bias was explored as a moderator to see whether effect sizes significantly differed across samples which had a higher risk of bias. The omnibus test with risk of bias was significant, *F*(1, 306) = 3.88, *p* = .050, $\tau_{\text{Level}\;2}^{2}$ = .01, $\tau_{\text{Level}\;3}^{2}$ = .01, *n* = 306, *k* = 67, *I* ^2^ = 81.74%, indicating that the overall relation between frequency of HMAs and mathematical skills is moderated by risk of bias, with studies reporting greater effects also showing higher risk of bias.

## DISCUSSION

This meta‐analysis provides the first synthesis of results and evaluation of study quality on the relation between the frequency of HMAs and mathematical skills in young children. The review brings much needed clarity to the research area as it suggests that children who engage in a higher frequency of HMAs tend to have higher mathematical skills than their peers who are engaged in a lower frequency of HMAs. The results of the meta‐analysis indicate that there is an overall small positive relation between the frequency of HMA and mathematical skills. The small effect (*r* = .13) may explain why there has been such a debate in the field regarding whether HMAs do genuinely support mathematical skills. The effect sizes varied between studies primarily due to true heterogeneity and not random error. Children's age, study design, geographical location of data collection, HMA measure type (overall, formal, informal), mathematical skill measure type, and publication bias did not significantly explain heterogeneity in effect size. Risk of bias did significantly moderate the effect such that studies higher in risk of bias showed a stronger relation between HMAs and children's mathematical skills.

The small relation observed raises questions around whether the frequency of HMAs is a beneficial target for intervention to improve early mathematical skills, as the small relation may indicate that, if this relation is causal, increasing the frequency of HMAs will result in minimal gains to early mathematical skills. Importantly, we found large variation in effect sizes not only between studies but also within studies. It is possible that the frequency of HMAs plays an important role in early mathematical skills only under certain circumstances or in certain populations, but not in others. Identifying factors that may increase or decrease the strength of this relation is, therefore, critical for directing future research and developing interventions in this area.

The risk of bias analysis revealed clues to the disparities in this literature's findings. Studies with a higher bias score were more likely to report an association between HMAs and children's mathematical skills. Studies scored higher in risk of bias if the sample size was small, the measures were less reliable and valid, there was a high level of task drop out on the mathematics assessments, and studies used listwise deletion to deal with missing data. All of the above factors can undermine a study's ability to detect a true effect size, thus meaning we can be less confident in the results of those studies (Button et al., 2013; Stahl & Pickles, 2017). It underscores the importance of studies being adequately powered, using valid and reliable measures that are age appropriate so less likely to have missing data and providing full reporting of these, including missing data. Results did not vary by mathematical skill, HMA type, age, study type, or geographical region.

We found that the type of mathematical skill measure (i.e., a composite set of skills, counting, number knowledge, magnitude knowledge, operations, or nonsymbolic measure) did not moderate the relation between HMAs and mathematical skills. Unfortunately, while we did code for spatial skills, only three samples measured spatial skills, and thus, we were unable to explore spatial skills as a subcategory of the maths skill moderator. The finding that HMAs do not uniquely predict individual mathematical skills is perhaps unsurprising given the multifaceted nature of many HMA questionnaires (e.g., Cahoon, Cassidy, et al., 2021; LeFevre et al., 2009). It may suggest that the HMA's asked about in questionnaires may have a broad, general influence on mathematical skills, rather than being specifically tied to specific mathematical skill types.

Similarly, the type of HMA measure used (an overall measure or formal and informal activities specifically) did not moderate the relation between HMAs and mathematical skills. In line with Mutaf‐Yıldız et al.'s (2020) review, we found that overall HMA measures were used the most, followed by formal HMA measures, with informal HMA distinction used the least. These findings are noteworthy, given that previous literature has claimed that formal activities may be more beneficial than informal ones for developing mathematical skills (Huntsinger et al., 2016; Lê & Noël, 2020). The results of the current review do not support this claim. We discuss the limitations of this divide further on in the discussion.

Additionally, study design (longitudinal vs. cross‐sectional) and the age of the child did not moderate the relation between frequency of HMAs and children's mathematical skills. These results replicated Daucourt et al.'s (2021) finding which examined the wider home mathematical environment. We might have expected relations to be stronger for younger children and over time, assuming that younger children need more parent input in learning which may lay the groundwork for developing mathematical skills. However, our review suggests no developmental effects to this relation.

Geographical location also did not moderate any effects. This was surprising, as we expected variation in results by geographical location due to access parents have to early‐years services and the age children begin school potentially shaping parenting home learning practices (Bertram et al., 2016; Organisation for Economic Co‐operation and Development, 2019). However, it is important to note that five independent samples fell outside of our three geographical regions. Hence, as more studies are conducted around the world, the role of geographical location in explaining variations of the strength of the relation between the frequency of HMAs and mathematical skills may change. However, careful measurement of a range of variables will be needed to disentangle effects of country from other important factors that may correlate, such SES, availability and quality of school provision, and cultural practices and parent attitudes to learning.

It is unfortunate that we were not able to examine SES due to limitations of how it was reported across studies (Psaki et al., 2014). Many papers did not report sample SES information, and many studies differ in their metric of SES (i.e., education or income or neighborhood measure; e.g., James‐Brabham et al., 2023; LeFevre et al., 2010; Skwarchuk et al., 2014). This made it difficult to compare SES indicators across studies, and, in particular, across countries. It is possible that children from higher‐SES families, who are more likely to have resources to support home mathematical learning, may engage in more HMAs from low‐SES families with fewer resources (Bradley & Corwyn, 2002), and this could explain why SES disparities in mathematical skills are visible even before children begin school (Blakey et al., 2020; James‐Brabham et al., 2023). It will be important moving forward for studies to aim to recruit socially diverse samples and to consistently report any SES characteristics of their samples (see Draper et al., 2022, for a discussion of this issue).

### Moving beyond frequency and questionnaires

The small overall effect found in the current meta‐analysis may indicate that when thinking about the best ways to support early mathematical development, focusing on the *frequency* of HMAs alone may not be particularly effective. This would perhaps suggest that future research should move away from exploring the frequency of HMAs for mathematical skills and instead endeavor to better understand the range and quality of activities that parents are doing in the home and how this interacts with children's abilities. Eason et al. (2022) reviews the multifaceted nature of the home mathematical environment, highlighting the reductionist nature of focusing solely on a single element of the home mathematical environment, and not understanding how different elements of the environment (e.g., resources, parent beliefs) may influence frequency and range and quality of activities. However, given the focus of the current review, we would like to highlight several ways in future studies can be improved if we are to better understand the role the home mathematical learning plays in early mathematical skills.

First, while all studies included in the current review measure the frequency that parents engage in HMAs with their children, there is little consensus in the literature about how HMAs should be measured, and as such, there is a wide variation of scales used (e.g., DeFlorio & Beliakoff, 2015; Lefevre et al., 2009, 2010). Variability in how HMAs are measured has been highlighted as a serious challenge to understanding how they relate to mathematical skills (Hornburg et al., 2021). Some scales may better capture the types of mathematical activities parents are doing with their children than others, leading to variation in effect sizes. Furthermore, the categorization of direct or formal and indirect or informal activities is inconsistent across studies, both in terms of the labels used and the method of categorizing. For instance, sometimes scale items are considered informal or formal based on their description (e.g., books considered formal; games informal), and sometimes, this is done statistically using factor analysis. This means sometimes similar items are categorized differently by studies and there is no consensus. Because of a lack of clear and consistent definitions of formal HMAs and informal HMAs, and studies often not reporting the questions used, we were unable to classify studies into formal or informal ourselves and instead had to rely on how authors had defined this. It is, therefore, likely that home mathematics activities overlap in both “formal” and “informal” scales across studies. Furthermore, measuring HMAs using a questionnaire provides little context to which activities took place, and therefore, we would argue that any categories such as “formal” or “informal” could be misguided. To illustrate, the item “counting objects” which appears on many HMA scales could be done in a formal or informal context, and without knowing this, it would be impossible to categorize this accurately (see Andrews et al., 2021, for a similar discussion). The limitations to the way formal and informal factors are derived limit the conclusions we can draw on this distinction in the current review.

A further limitation of existing research using HMA scales is that often, it is unclear how scales have been developed and what reliability checks were conducted, if any. Few studies have used a frequency‐based HMA questionnaire which was rigorously developed with input from parents (for exceptions, see Cahoon, Cassidy, et al., 2021; Cosso et al., 2024). More work needs to be done in developing reliable and valid scales that have been co‐developed with parents if we are to better understand the role frequency of home mathematical activities play in mathematical skills.

If we want to understand how HMAs support mathematical skills using existing questionnaire data, one suggestion is to group by the mathematical skills targeted by each activity (rather than inferring formal or informal classifications). For example, questionnaires frequently ask about counting objects, as well as weighing out ingredients, or telling the time (e.g., Cahoon, Cassidy, et al., 2021; Cahoon, Gilmore, et al., 2021; DeFlorio & Beliakoff, 2015; Lefevre et al., 2009). It might be anticipated that these questions tap into different mathematical skills, for example, counting objects may support cardinal principal development, whereas weighing out objects may be more likely to support numerical magnitude skills. It would not necessarily be expected that telling the time would relate to nonsymbolic number knowledge. By aggregating the frequency scores of these activities, either by averaging together (e.g., Dearing et al., 2012; Zippert & Ramani, 2017) or by dividing into subscales such as formal versus informal (e.g., DeFlorio & Beliakoff, 2015; Lefevre et al., 2009), sensitivity may be lost to detect how these activities influence mathematical skills. Therefore, to better understand the role of frequency of HMAs, it is important to move toward understanding how mathematical activities which may nurture specific mathematical skills relate to said mathematical skill (see also Andrews et al., 2021).

Continuing in the same vein, addressing the limitations surrounding the assessment of HMAs is crucial. It is possible that self‐reports of HMA frequency do not accurately capture the actual frequency of HMAs taking place, which is supported by research comparing questionnaire data with semi‐structured interview data on HMAs which found they did not correlate with one another (Mutaf Yildiz et al., 2018). This discrepancy may arise because often parents do not immediately realize that many of their learning interactions involve mathematical skills (see discussion in Hornburg et al., 2021). This is problematic for relying on the findings of the current literature to draw wider conclusions about the role of HMAs as it may be those self‐report questionnaires simply do not adequately capture the frequency that parents are engaging in specific HMAs with their children, let alone the range or quality of those activities. We suggest that to gain a more meaningful understanding of the role of HMAs, it is important that future research moves beyond questionnaires. Measures more suitable for capturing the types of mathematical activities taking place include observational data recorded in the home either directly through video or voice recordings or indirectly through diaries and interviews. Susperreguy and Davis‐Kean (2016) provide a good example of how home mathematical interactions can be measured naturalistically via recordings. They recorded maternal mathematical talk during family mealtimes and found it related to children's later mathematical attainment. More work using this approach to capture not just the frequency but also the range and quality of HMAs will be important going forward in understanding the key features of HMAs that really count.

Additionally, it is important to emphasize that correlation does not equal causation. Intervention research used as a methodological tool to test causal relations seems a crucial next step. To be clear: we are not suggesting as a next step that applied interventions would improve children's mathematical skills. The fact that we find a small effect size suggests additional rigorous research exploring this relation with robust methods is needed. Specifically, we suggest that interventions used as a methodological tool may help to shed light on the relation between specific mathematical activities (e.g., counting‐based activities) on specific mathematical abilities which arise in the early years (e.g., counting skills and cardinal principal knowledge).

## CONCLUSIONS

The present meta‐analysis is the first quantitative synthesis of the relation between the frequency of HMAs and mathematical skills in young children. Although the strength and direction of effect sizes vary widely between and within studies, this meta‐analysis found an overall small positive relation between the frequency of HMAs and mathematical skills. While our findings suggest that the frequency of HMAs has only a small relation with mathematical skills, caution is warranted before drawing definitive conclusions because of the risk of bias apparent in many studies. We have discussed several limitations within the field that must be addressed. First, the accurate measurement of home mathematical skills remains a challenge, and the quality and range of these activities are often overlooked. Second, the issue of risk of bias within existing studies necessitates scrutiny. Therefore, while it may appear that increasing the frequency of HMAs would minimal benefits for mathematical skills, if found to be causal, based on our analysis, ongoing research efforts and improved methodological rigor are essential before reaching any definitive conclusions about the relation between frequency of HMAs and mathematical skills.

## AUTHOR CONTRIBUTIONS

Ella James‐Brabham: Conceptualization, data curation, formal analysis, investigation, methodology, project administration, writing—original draft, writing—review and editing. Claudia C. von Bastian: Methodology, writing—review and editing. Carmel Brough: Data curation, writing—review and editing. Emma Blakey: Conceptualization, methodology, project administration, supervision, writing—review and editing.

## Supporting information


Appendix S1.

## Data Availability

Data from this study are available in Supporting Information. The analytic code is available from the corresponding author.
