# Supplementary material for: Do home mathematical activities relate to early mathematical skills? A systematic review and meta‐analysis
Source: Child Dev. 2024 Oct 3;96(1):451–68. doi: 10.1111/cdev.14162 (PMC11693824; doi:10.1111/cdev.14162)
Supplement: Supplementary file 1 — Appendix S1. [file CDEV-96-451-s001.zip › S3.docx]

| **Country** | ***r*** | **95% CI** | ***n*** | ***k*** |
| --- | --- | --- | --- | --- |
| United States | .14 | 0.09, 0.19 | 83 | 24 |
| Canada | .13 | 0.03, 0.24 | 22 | 6 |
| Belgium | .13 | 0.02, 0.24 | 20 | 4 |
| Germany | .12 | 0.02, 0.22 | 14 | 5 |
| United Kingdom | .10 | 0.02, 0.18 | 46 | 8 |
| Chile | .06 | -0.09, 0.20 | 22 | 2 |
| China | .11 | 0.00, 0.23 | 35 | 3 |
| Hong Kong | .12 | -0.02, 0.26 | 24 | 2 |
| Philippines | .09 | -0.05, 0.23 | 12 | 2 |

**S3**

### **Moderation Effects of Country of Data Collection (*k* = 56)**

The omnibus test with country of data collection was not significant, *F*[8, 269] = 0.24, *p* = .984, , τ^2^_Level 2_ = 0.00_,_ τ^2^_Level3_ = 0.01, *n* = 278, *I*^2^ = 77.72%, indicating that the overall relation between frequency of HMA and mathematical skills was not significantly moderated by country.

Note. *r* = Pearson’s *r*, CI = confidence interval, *n* = number of effect sizes, *k* = number of independent samples.
